# A case of condyloma acuminatum of the bladder concurrently diagnosed with urothelial carcinoma

**DOI:** 10.1002/iju5.12664

**Published:** 2023-11-08

**Authors:** Ibuki Tsuru, Masaki Nakamura, Taro Izumi, Akihiro Ono, Sakiko Miura, Teppei Morikawa, Kazuyoshi Shigehara, Tadaichi Kitamura, Haruki Kume, Yoshiyuki Shiga

**Affiliations:** ^1^ Department of Urology NTT Medical Center Tokyo Tokyo Japan; ^2^ Department of Diagnostic Pathology NTT Medical Center Tokyo Tokyo Japan; ^3^ Department of Integrative Cancer Therapy and Urology Kanazawa University Graduate School of Medical Science Kanazawa Japan; ^4^ Japanese Foundation for Sexual Health Medicine Tokyo Japan; ^5^ Department of Urology The University of Tokyo hospital Tokyo Japan

**Keywords:** bladder, condyloma acuminatum, human papilloma virus, urothelial carcinoma

## Abstract

**Introduction:**

Condyloma acuminatum usually occurs in the external genitalia and rarely in the bladder mucosa. Here, we report a case of condyloma acuminatum of the bladder that was detected concurrently with urothelial carcinoma.

**Case presentation:**

A 42‐year‐old man was referred to our urology department with positive urine cytology for urothelial carcinoma. Cystoscopy revealed a broad‐base nonpapillary bladder tumor. The patient underwent a transurethral resection of the bladder tumor. Pathological examination revealed urothelial carcinoma, high‐grade pT1, and concurrent resection of condyloma acuminatum. DNA was extracted from the paraffin‐embedded transurethral resection of the bladder tumor tissue specimens. HPV11 was detected in condylomas by PCR and in situ hybridization, whereas HPV was not detected in urothelial carcinomas.

**Conclusion:**

We report a rare case of condyloma acuminatum of the bladder that was concurrently diagnosed with urothelial carcinoma from the same site.

**Abbreviations & Acronyms**
BCG: Bacillus Calmette–Guérin
HPV: human papillomavirus
SCC: squamous cell carcinoma
TUR‐Bt: transurethral resection of the bladder tumor

**Keynote message**

In addition to being rare, condyloma acuminatum is associated with a high risk of malignancy. Moreover, the association of condyloma acuminatum with bladder cancer has been controversial. Here, we report a rare case of condyloma acuminatum of the bladder that was concurrently diagnosed with urothelial carcinoma at the same site.

## Introduction

Condyloma acuminatum is a HPV infection resulting in the formation of a wart, and it is relatively common among young adults. The annual incidence of condyloma acuminatum ranges from 160 in Spain to 289 in the United Kingdom cases per 100 000 individuals.^1^

The most common sites affected by condyloma acuminatum are the perianal area, perineum, labia, penis, anal canal, and vagina, and it rarely occurs in the bladder. Herein, we report a case of bladder condyloma acuminatum concurrently diagnosed with bladder cancer from the same site.

## Case

**Fig. 1:**
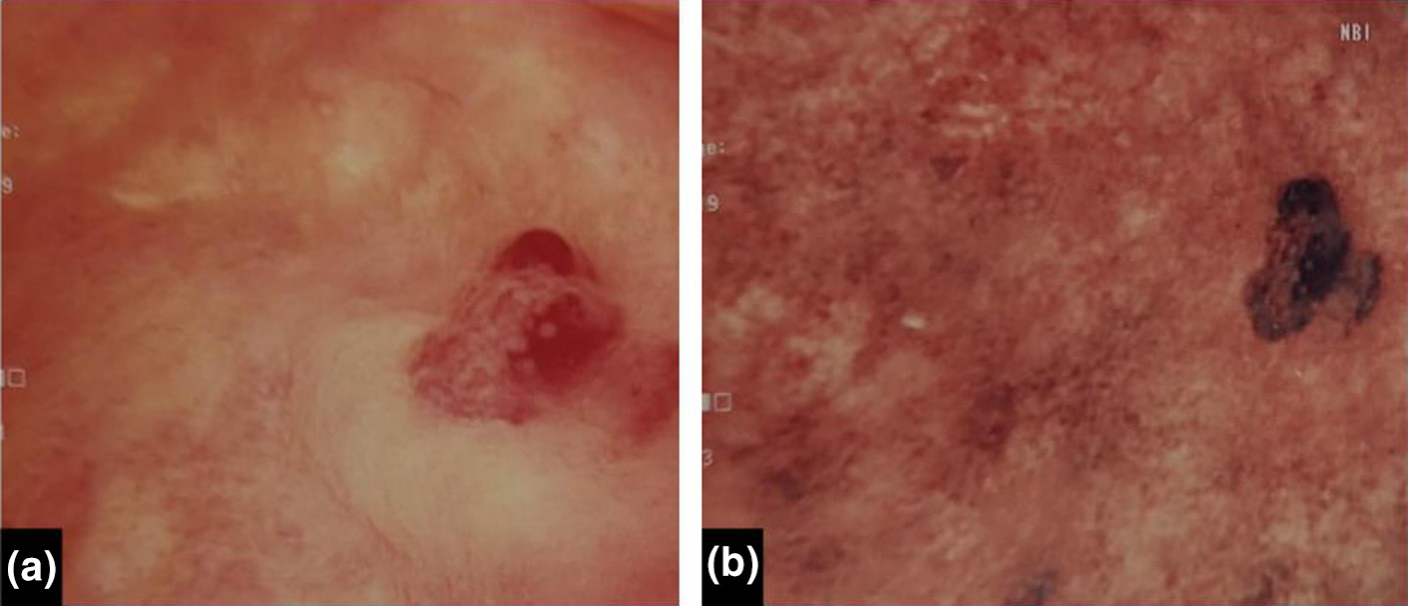
(a) Cystoscopy of the broad‐base non‐papillary bladder tumor. (b) Narrow band imaging of the bladder tumor.

A 42‐year‐old male presented to a nearby clinic complaining of macroscopic hematuria and pain during urination. The patient was in good condition and had no urinary infection. Urine cytology was positive for urothelial carcinoma, and the patient was referred to our hospital. The patient had no history of HPV infection but he had multiple heterosexual partners. The physical and laboratory findings were within normal limits. The patient was immunocompetent and was free from HIV infection. Cystoscopy revealed a broad‐base nonpapillary bladder tumor (Fig. 1a,b). There were no specific findings in the urethra. Computed tomography examination showed no apparent metastasis of the bladder tumor.

**Fig. 2:**
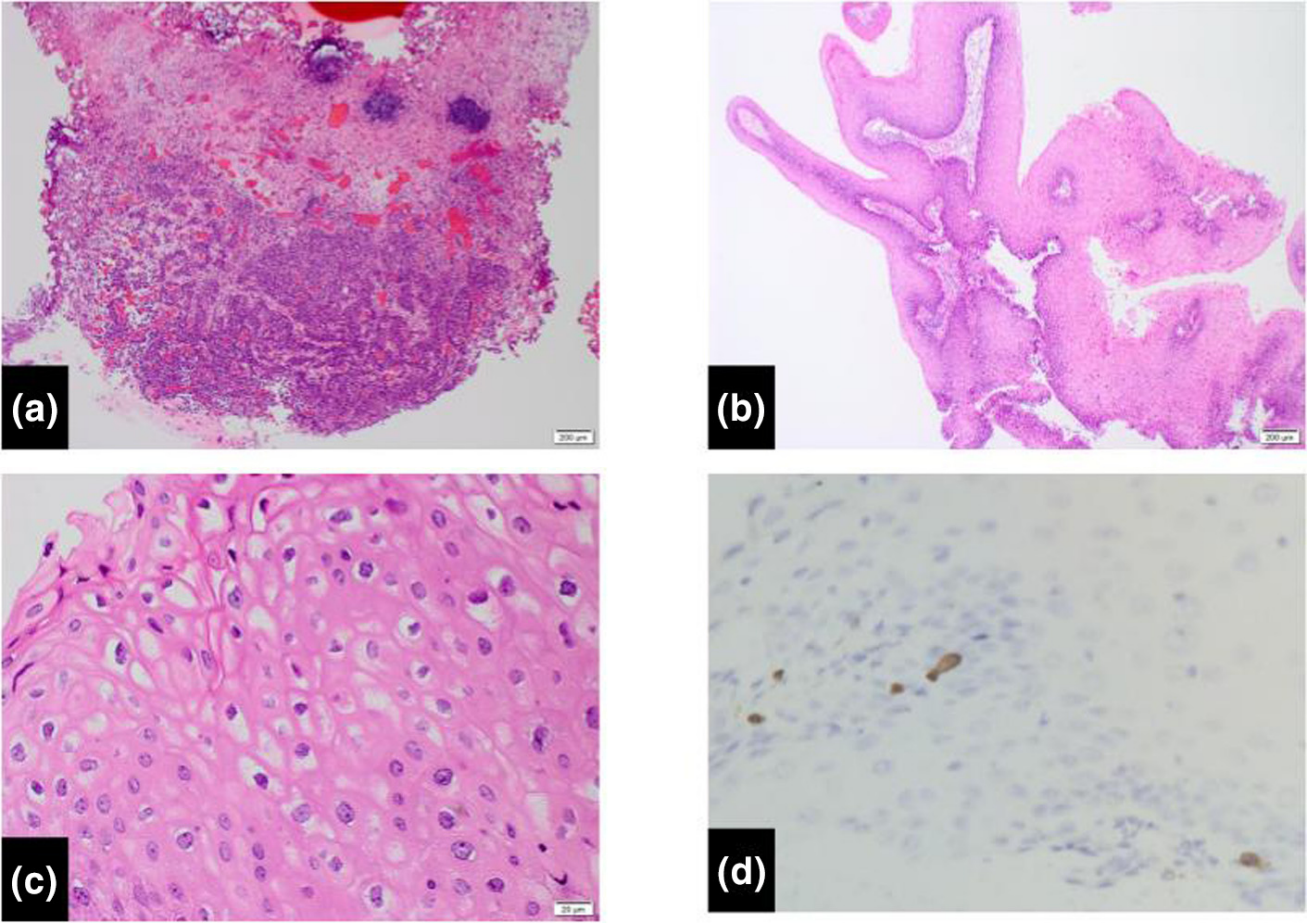
(a) Urothelial carcinoma. (b) Condyloma acuminatum. Papillary proliferation of squamous epithelium is observed (4×). (c) Koilocytosis of the condyloma acuminatum (40×). (d) In situ hybridization signals for HPV DNA of condyloma acuminatum.

Transurethral resection of the bladder tumor was performed, and histopathologic examination revealed high‐grade, pT1 urothelial carcinoma (Fig. 2a). The resected specimen concurrently involved the condyloma acuminatum of the bladder. There was also a poorly dysmorphic, stratified squamous epithelium and a papillary lesion covered with a proliferating squamous epithelium. Binucleated cells and koilocytosis were observed, which may correspond to condyloma acuminatum (Fig. 2b,c). We extracted from the paraffin‐embedded TUR‐Bt tissue specimens. HPV11 was detected in condyloma using PCR and in situ hybridization, whereas HPV was not detected in urothelial carcinoma (Fig. 2d).

The patient underwent 7 weeks of intravesical instillation of BCG. The second TUR‐Bt was performed 3 months after the first cycle of BCG therapy. We did not detect residual urothelial carcinoma or condyloma acuminatum. However, about 6 months later, cystoscopy revealed mucosal irregularities and redness, and urine cytology was positive for urothelial carcinoma. The patient underwent TUR‐Bt and the pathological diagnosis was high‐grade urothelial carcinoma in situ. No recurrence of condyloma acuminatum was observed. We recommended radical cystectomy for a BCG refractory case, but the patient wished to avoid radical cystectomy and preserve sexual function. Therefore, we performed a second BCG intravesical instillation therapy. The patient is currently under careful follow‐up.

## Discussion

Condyloma acuminatum is relatively common but rarely involves the urethra or bladder.^2^ Condyloma acuminatum is attributed to HPV infection. HPV types 6 and 11 account for most condyloma acuminatum cases.

Given the route of HPV transmission, sexual contact results in an HPV infection affecting the external genitalia. Moreover, the infection invades the urothelial epithelium of the bladder through the urethra.^3^ The occurrence of condyloma acuminatum of the urinary tract has been reported in immunosuppressive conditions, such as renal transplant recipient.^4^ In this case, he had multiple heterosexual partners, which could be considered a risk factor, but there were no prior sexually transmitted infections or urethritis. The route of infection was unknown; however, a subclinical HPV infection was suspected.

Condyloma acuminatum has malignant potential. HPV can infect the cervix, oropharynx, anus, penis, vagina, and vulva. Worldwide, high‐risk HPV covers 10% of all cancers, with an estimated 570 000 women and 60 000 men with HPV‐related cancer yearly.^5^ HPV 16 positive bladder carcinoma was first reported in 1988, but the causal relationship between HPV and bladder cancer has been controversial.^6^, ^7^, ^8^, ^9^, ^10^, ^11^, ^12^, ^13^ A previous meta‐analysis showed a significantly high association between HPV infection and bladder cancer, with HPV‐16 being the most prevalent genotype in bladder cancer.^14^ On the other hand, two recent meta‐analyses have shown conflicting results, one showing that HPV infection is not associated with bladder cancer (OR: 2.077, 95% CI: 0.940–4.587),^6^ while the other reported a significant association between HPV infection and bladder cancer (OR: 7.8406, 95% CI: 4.343–14.16).^15^

However, condyloma acuminatum can develop into SCC. Samarska *et al*. summarized 38 patients with condyloma acuminatum of the bladder, 17 of whom had SCC concurrently with condyloma acuminatum or within a year of diagnosis.^16^ Although low‐risk HPV (HPV 6/11) is known to have little association with SCC at head and neck, gynecology tract, and anal cancers, the study showed an association of condyloma acuminatum and SCC with low‐risk HPV and high‐risk HPV.^16^ Condyloma acuminatum of the bladder is associated with SCC of the bladder, regardless of the HPV subtype. The high affinity of HPV to differentiating squamous epithelium and the ability to evade immune responses may explain the mechanism of cancer development in the bladder epithelium.^15^

A meta‐analysis demonstrated that the association between HPV and bladder cancer risk was highest among Asians (OR 6.289; 95% CI 2.167–18.250).^6^ In addition to geographical factors, age is also a risk factor for HPV‐related cancer. Among bladder cancer patients, HPV‐positive patients were younger than HPV‐negative patients, with mean ages of 60.2 and 70.3 years, respectively.^17^ In this case, the direct relationship between condyloma acuminatum and urothelial carcinoma is unclear; however, Asian ethnicity and young age are consistent risk factors.

## Conclusion

We report a rare case of condyloma acuminatum of the bladder that was concurrently diagnosed with urothelial carcinoma at the same site. We detected HPV DNA in condyloma acuminatum samples, but HPV DNA was not in urothelial carcinoma samples. No consensus exists on the surveillance of condyloma acuminatum of the bladder. Therefore, close and careful observation is necessary.

## Author contributions

Ibuki Tsuru: Writing – original draft. Masaki Nakamura: Supervision; writing – original draft; writing – review and editing. Taro Izumi: Writing – review and editing. Akihiro Ono: Writing – review and editing. Sakiko Miura: Writing – review and editing. Teppei Morikawa: Writing – review and editing. Kazuyoshi Shigehara: Investigation; resources; writing – review and editing. Tadaichi Kitamura: Writing – review and editing. Haruki Kume: Writing – review and editing. Yoshiyuki Shiga: Supervision.

## Conflict of interest

None of the contributing authors have any conflicts of interest.

## Approval of the research protocol by an Institutional Reviewer Board

Not applicable.

## Informed consent

Written informed consent for publication was obtained from the patient.

## Registry and the Registration No. of the study/trial

Not applicable.

## References

[1] Patel H , Wagner M , Singhal P , Kothari S . Systematic review of the incidence and prevalence of genital warts. BMC Infect. Dis. 2013; 13: 39. 23347441 10.1186/1471-2334-13-39 PMC3618302

[2] Murray AJ , Bivalacqua TJ , Sopko NA . Innumerable condyloma acuminatum tumors of the bladder. Urol. Case Rep. 2017; 12: 76–77. 28377892 10.1016/j.eucr.2016.10.006 PMC5377425

[3] Shigehara K , Sasagawa T , Namiki M . Human papillomavirus infection and pathogenesis in urothelial cells: a mini‐review. J. Infect. Chemother. 2014; 20: 741–747. 25271131 10.1016/j.jiac.2014.08.033

[4] Sarier M , Ozel E , Duman I , Yuksel Y , Demirbas A . HPV type 45‐positive condyloma acuminata of the bladder in a renal transplant recipient. Transpl. Infect. Dis. 2017; 19: e12667. 10.1111/tid.12667 28100036

[5] de Martel C , Plummer M , Vignat J , Franceschi S . Worldwide burden of cancer attributable to HPV by site, country and HPV type. Int. J. Cancer 2017; 141: 664–670. 28369882 10.1002/ijc.30716 PMC5520228

[6] Khatami A , Salavatiha Z , Razizadeh MH . Bladder cancer and human papillomavirus association: a systematic review and meta‐analysis. Infect. Agent Cancer 2022; 17: 3. 35062986 10.1186/s13027-022-00415-5 PMC8780707

[7] Youshya S , Purdie K , Breuer J *et al*. Does human papillomavirus play a role in the development of bladder transitional cell carcinoma? A comparison of PCR and immunohistochemical analysis. J. Clin. Pathol. 2005; 58: 207–210. 15677544 10.1136/jcp.2004.017152 PMC1770580

[8] Helal Tel A , Fadel MT , El‐Sayed NK . Human papilloma virus and p53 expression in bladder cancer in Egypt: relationship to schistosomiasis and clinicopathologic factors. Pathol. Oncol. Res. 2006; 12: 173–178. 16998598 10.1007/BF02893365

[9] Lopez‐Beltran A , Escudero AL , Vicioso L , Muñoz E , Carrasco JC . Human papillomavirus DNA as a factor determining the survival of bladder cancer patients. Br. J. Cancer 1996; 73: 124–127. 8554974 10.1038/bjc.1996.23 PMC2074275

[10] Sano T , Sakurai S , Fukuda T , Nakajima T . Unsuccessful effort to detect human papillomavirus DNA in urinary bladder cancers by the polymerase chain reaction and in situ hybridization. Pathol. Int. 1995; 45: 506–512. 7551011 10.1111/j.1440-1827.1995.tb03493.x

[11] Gutiérrez J , Jiménez A , de Dios Luna J , Soto MJ , Sorlózano A . Meta‐analysis of studies analyzing the relationship between bladder cancer and infection by human papillomavirus. J. Urol. 2006; 176: 2474–2481. 17085133 10.1016/j.juro.2006.07.157

[12] Griffiths TR , Mellon JK . Human papillomavirus and urological tumours: II. Role in bladder, prostate, renal and testicular cancer. BJU Int. 2000; 85: 211–217. 10671869 10.1046/j.1464-410x.2000.00465.x

[13] Kitamura T , Yogo Y , Ueki T , Murakami S , Aso Y . Presence of human papillomavirus type 16 genome in bladder carcinoma in situ of a patient with mild immunodeficiency. Cancer Res. 1988; 48: 7207–7211. 2847865

[14] Li N , Lin Y , Yawei Z , Ping Z , Tongzhang Z , Min D . Human papillomavirus infection and bladder cancer risk: a meta‐analysis. J Infect Dis 2011; 204: 217–223. 21673031 10.1093/infdis/jir248 PMC3114469

[15] Muresu N , Di Lorenzo B , Saderi L *et al*. Prevalence of human papilloma virus infection in bladder cancer: a systematic review. Diagnostics 2022; 12: 1759. 35885662 10.3390/diagnostics12071759 PMC9318826

[16] Samarska IV , Epstein JI . Condyloma acuminatum of urinary bladder: relation to squamous cell carcinoma. Am. J. Surg. Pathol. 2019; 43: 1547–1553. 31368913 10.1097/PAS.0000000000001339

[17] Shigehara K , Sasagawa T , Kawaguchi S *et al*. Etiologic role of human papillomavirus infection in bladder carcinoma. Cancer 2011; 117: 2067–2076. 21523718 10.1002/cncr.25777

